# Population Enumeration and Household Utilization Survey Methods in the Enterics for Global Health (EFGH): *Shigella* Surveillance Study

**DOI:** 10.1093/ofid/ofae018

**Published:** 2024-03-25

**Authors:** Ryan Dodd, Alex O Awuor, Paul F Garcia Bardales, Farhana Khanam, Donnie Mategula, Uma Onwuchekwa, Golam Sarwar, Mohammad Tahir Yousafzai, Naveed Ahmed, Hannah E Atlas, Md Amirul Islam Bhuiyan, Josh M Colston, Bakary Conteh, Manan Diawara, Nasrin Dilruba, Sarah Elwood, Irum Fatima, Erika Feutz, Sean R Galagan, Shahinur Haque, Md Taufiqul Islam, Mehrab Karim, Belali Keita, Margaret N Kosek, Karen L Kotloff, Clement Lefu, Mamadou Mballow, Maureen Ndalama, Latif Ndeketa, Billy Ogwel, Caleb Okonji, Maribel Paredes Olortegui, Patricia B Pavlinac, Tackeshy Pinedo Vasquez, James A Platts-Mills, Firdausi Qadri, Sonia Qureshi, Elizabeth T Rogawski McQuade, Shazia Sultana, Moussa Oumar Traore, Nigel A Cunliffe, M Jahangir Hossain, Richard Omore, Farah Naz Qamar, Milagritos D Tapia, Pablo Peñataro Yori, K Zaman, Christine J McGrath

**Affiliations:** Division of Infectious Diseases and International Health, School of Medicine, University of Virginia, Charlottesville, Virginia, USA; Kenya Medical Research Institute, Center for Global Health Research (KEMRI-CGHR), Kisumu, Kenya; Asociación Benéfica PRISMA, Iquitos, Peru; Infectious Diseases Division, International Centre for Diarrhoeal Disease Research, Bangladesh, Dhaka, Bangladesh; Malawi Liverpool Wellcome Trust Clinical Research Programme, Blantyre, Malawi; Department of Health Systems and Policy, Kamuzu University of Health Sciences, School of Global Public Health, Blantyre, Malawi; Liverpool School of Tropical Medicine, Liverpool, United Kingdom; Centre Pour le Développement des Vaccins du Mali (CVD-Mali), Bamako, Mali; Medical Research Council Unit The Gambia, London School of Hygiene and Tropical Medicine, Fajara, The Gambia; Department of Pediatrics and Child Health, The Aga Khan University, Karachi, Pakistan; Department of Pediatrics and Child Health, The Aga Khan University, Karachi, Pakistan; Department of Global Health, University of Washington, Seattle, Washington, USA; Infectious Diseases Division, International Centre for Diarrhoeal Disease Research, Bangladesh, Dhaka, Bangladesh; Division of Infectious Diseases and International Health, School of Medicine, University of Virginia, Charlottesville, Virginia, USA; Medical Research Council Unit The Gambia, London School of Hygiene and Tropical Medicine, Fajara, The Gambia; Centre Pour le Développement des Vaccins du Mali (CVD-Mali), Bamako, Mali; Center for Vaccine Development and Global Health, University of Maryland School of Medicine, Baltimore, Maryland, USA; Division of Infectious Diseases and International Health, School of Medicine, University of Virginia, Charlottesville, Virginia, USA; Department of Pediatrics and Child Health, The Aga Khan University, Karachi, Pakistan; Department of Global Health, University of Washington, Seattle, Washington, USA; Department of Global Health, University of Washington, Seattle, Washington, USA; Infectious Diseases Division, International Centre for Diarrhoeal Disease Research, Bangladesh, Dhaka, Bangladesh; Infectious Diseases Division, International Centre for Diarrhoeal Disease Research, Bangladesh, Dhaka, Bangladesh; Medical Research Council Unit The Gambia, London School of Hygiene and Tropical Medicine, Fajara, The Gambia; Medical Research Council Unit The Gambia, London School of Hygiene and Tropical Medicine, Fajara, The Gambia; Division of Infectious Diseases and International Health, School of Medicine, University of Virginia, Charlottesville, Virginia, USA; Center for Vaccine Development and Global Health, University of Maryland School of Medicine, Baltimore, Maryland, USA; Department of Pediatrics, University of Maryland School of Medicine, Baltimore, Maryland, USA; Department of Medicine, University of Maryland School of Medicine, Baltimore, Maryland, USA; Malawi Liverpool Wellcome Trust Clinical Research Programme, Blantyre, Malawi; Medical Research Council Unit The Gambia, London School of Hygiene and Tropical Medicine, Fajara, The Gambia; Malawi Liverpool Wellcome Trust Clinical Research Programme, Blantyre, Malawi; Malawi Liverpool Wellcome Trust Clinical Research Programme, Blantyre, Malawi; Department of Health Systems and Policy, Kamuzu University of Health Sciences, School of Global Public Health, Blantyre, Malawi; Institute of Infection, Veterinary and Ecological Sciences, University of Liverpool, Liverpool, United Kingdom; Kenya Medical Research Institute, Center for Global Health Research (KEMRI-CGHR), Kisumu, Kenya; Kenya Medical Research Institute, Center for Global Health Research (KEMRI-CGHR), Kisumu, Kenya; Asociación Benéfica PRISMA, Iquitos, Peru; Department of Global Health, University of Washington, Seattle, Washington, USA; Asociación Benéfica PRISMA, Iquitos, Peru; Division of Infectious Diseases and International Health, School of Medicine, University of Virginia, Charlottesville, Virginia, USA; Infectious Diseases Division, International Centre for Diarrhoeal Disease Research, Bangladesh, Dhaka, Bangladesh; Department of Pediatrics and Child Health, The Aga Khan University, Karachi, Pakistan; Department of Epidemiology, Emory University, Atlanta, Georgia, USA; Department of Pediatrics and Child Health, The Aga Khan University, Karachi, Pakistan; Centre Pour le Développement des Vaccins du Mali (CVD-Mali), Bamako, Mali; Institute of Infection, Veterinary and Ecological Sciences, University of Liverpool, Liverpool, United Kingdom; Medical Research Council Unit The Gambia, London School of Hygiene and Tropical Medicine, Fajara, The Gambia; Kenya Medical Research Institute, Center for Global Health Research (KEMRI-CGHR), Kisumu, Kenya; Department of Pediatrics and Child Health, The Aga Khan University, Karachi, Pakistan; Center for Vaccine Development and Global Health, University of Maryland School of Medicine, Baltimore, Maryland, USA; Department of Pediatrics, University of Maryland School of Medicine, Baltimore, Maryland, USA; Department of Medicine, University of Maryland School of Medicine, Baltimore, Maryland, USA; Division of Infectious Diseases and International Health, School of Medicine, University of Virginia, Charlottesville, Virginia, USA; Infectious Diseases Division, International Centre for Diarrhoeal Disease Research, Bangladesh, Dhaka, Bangladesh; Department of Global Health, University of Washington, Seattle, Washington, USA

**Keywords:** EFGH, healthcare utilization survey, hybrid surveillance design, population enumeration, *Shigella*

## Abstract

**Background:**

Accurate estimation of diarrhea incidence from facility-based surveillance requires estimating the population at risk and accounting for case patients who do not seek care. The Enterics for Global Health (EFGH) *Shigella* surveillance study will characterize population denominators and healthcare-seeking behavior proportions to calculate incidence rates of *Shigella* diarrhea in children aged 6–35 months across 7 sites in Africa, Asia, and Latin America.

**Methods:**

The Enterics for Global Health (EFGH) *Shigella* surveillance study will use a hybrid surveillance design, supplementing facility-based surveillance with population-based surveys to estimate population size and the proportion of children with diarrhea brought for care at EFGH health facilities. Continuous data collection over a 24 month period captures seasonality and ensures representative sampling of the population at risk during the period of facility-based enrollments. Study catchment areas are broken into randomized clusters, each sized to be feasibly enumerated by individual field teams.

**Conclusions:**

The methods presented herein aim to minimize the challenges associated with hybrid surveillance, such as poor parity between survey area coverage and facility coverage, population fluctuations, seasonal variability, and adjustments to care-seeking behavior.

## Teaser

The Enterics for Global Health (EFGH): *Shigella* Surveillance Study will utilize a hybrid surveillance design to characterize population denominators and healthcare-seeking behavior to calculate *Shigella* diarrhea incidence rates in children aged 6 to 35 months across sites in Africa, Asia, and Latin America.

**Key Points**

The Enterics for Global Health (EFGH): *Shigella* Surveillance Study will use a hybrid surveillance design to characterize population denominators and healthcare-seeking behavior and calculate *Shigella* diarrhea incidence rates in children aged 6–35 months in Africa, Asia, and Latin America.

Surveillance of *Shigella-*attributable diarrhea is essential for monitoring disease burden and setting national and local priorities for the implementation and evaluation of public health interventions. However, there are limited data on *Shigella* incidence in low- and middle-income countries. The adoption of newly licensed *Shigella* vaccines into national immunization programs will require up-to-date country-specific data on the burden of *Shigella-*associated diarrhea and an understanding of the health and economic consequences of infection. The Enterics for Global Health (EFGH) *Shigella* surveillance study will use a hybrid surveillance design that uses gridded population estimation informed sampling to establish the denominator for calculating incidence rates of medically-attended diarrhea.

When *Shigella* cases are identified at facilities, estimates of population size are crucial to calculating *Shigella*-attributed diarrhea incidence, as this provides the denominator or underlying population at-risk from which cases arise. Common approaches to obtaining population estimates include a census, gridded population estimates based on satellite and census data, and surveying a sample of the population. A census enumerates the entire population and is the most accurate method for estimating the population at risk [1]. However, conducting a census is expensive, resource intensive, and time consuming, and a census may not capture current population dynamics if conducted infrequently [2]. More recently, gridded population estimations that incorporate satellite images and census data, such as WorldPop [3], have been used to provide population estimates [4, 5].

However, these estimates are limited by underlying data quality and cannot account for rapid population changes [4, 6]. Survey sampling uses data from a subset of the population to estimate characteristics of the entire population and is less resource intensive and efficient than a census [1]. Sampling can be enhanced by obtaining existing remotely generated information on population density, making the sampling process more logistically feasible for field teams. Case ascertainment through health facility–based surveillance is practical because it means that staff and supplies can be centralized in these facilities, but facility-based surveillance misses case patients who do not seek care, as diarrhea is often mild and self-limiting [7–9]. A healthcare-seeking adjustment to calculated incidence rates, ideally collected through healthcare utilization surveys (HUSs), can account for those cases missed by facility-based surveillance. Adjusted incidence rates, calculated through this hybrid surveillance design, enhance data accuracy, informing vaccine trial design and improving public health outcomes [2].

The Enterics for Global Health (EFGH) *Shigella* surveillance study will use a hybrid surveillance approach to estimate the incidence of *Shigella*-attributed diarrhea in children aged 6–35 months in 7 country sites in Africa, Asia, and South America. Underlying population size will be based on sampling all households within randomly selected clusters in each EFGH health facility catchment area. This cluster-based sampling approach will enable the comparison of sample-based population estimates to WorldPop estimates, which, if similar, could provide a pragmatic and inexpensive solution to enumerating catchment areas in future diarrhea incidence surveillance activities. HUSs will be administered to establish the proportion of children with diarrhea of various severities brought for care at EFGH health facilities. Population enumeration and HUSs will be performed continuously over a 24 month period alongside the diarrhea case surveillance at EFGH health facilities in the 7 country sites. This continuous data collection will ensure that seasonal changes in the underlying population and healthcare-seeking behavior is captured to accurately derive adjusted *Shigella* diarrhea incidence estimates in EFGH catchment areas.

## METHODS

### Catchment Areas

Catchment area descriptions are provided in Table 1. The principles for catchment area selection were (1) a sufficiently large target population and *Shigella* incidence to meet enrollment targets; (2) reasonably well-defined catchment area for the health facilities where diarrhea surveillance is to be conducted; and (3) a catchment area for each participating health facility that can be defined by ≥1 administrative unit (eg, ward, village, area, health service unit, demographic surveillance system area, township, or village), which can be used to clearly determine, during population enumeration and diarrhea surveillance activities, whether a given child or family lives within the residential area.

**Table 1.** Enterics for Global Health Study Area and Estimated Catchment Population by Study Site

Site	City	Administrative Unit	Area Size, km^2^	WorldPop Population, No.^a^		No. of Health Facilities	Health Facilities
				Total	Age <5 y		
Bangladesh	Dhaka South City Corporation	Ward	5.01	327 624	21 435	5	International Centre for Diarrhoeal Disease Research Dhaka Hospital, Enterics for Global Health Field Office, Mugda Medical College Hospital, Dhaka Medical College Hospital, Sir Salimullah Medical College Hospital
Kenya	Siaya County	Village	1146.84	519 549	78 478	9	Siaya County Referral Hospital, Lwak Mission Hospital, Abidha Health Center, Ongielo Health Centre, Akala Health Center, Wagai Health Center, Dienya Health Centre, Ting Wangi Health Center, Bar Agulu Health Center
Malawi	Blantyre City	Health surveillance assistant catchment area	6.31	54 071	7518	1	Ndirande Health Centre
Mali	Bamako	Quartier	7.44	250 013	35 829	4	Banconi Centres de Sante Communautaire, Asacodjeneka Centres de Sante, Asacodjip Centres de Sante, CSREF Commune 1
Pakistan	Karachi	Area (colony)	46.35	1 339 242	124 022	6	Ali Akbar Shah Vital Pakistan Trust Centre, Khidmat-e-Alam Medical Centre, Bhains Colony Vital Pakistan Trust Centre, Abbasi Shaheed Hospital, Sindh Government Hospital, Karongi, Sindh Government Hospital, Ibrahim Hyderi
Peru	Iquitos	Health insurance area	28.36	98 223^b^	3 824^b^	5	America, San Juan, Progreso, Modelo, Santo Tomas
The Gambia	Upper River Division	Village	696.39	145 854	28 986	2	Basse Hospital, Gambisara Health Centre

Abbreviation: CSREF, Centres de Santé Référence.

^a^WorldPop population estimations are derived by summing the estimated population of every cluster in each sites catchment area.

^b^A correction factor of 3 has been applied to WorldPop's population estimation because we believe it underestimates the population in and around Iquitos (which is highly mobile and prone to seasonal migration) by a factor of 3, based on a comparison between WorldPop's estimation of population of the city and official government population figures. (https://www.gob.pe/institucion/inei/informes-publicaciones/3464927-peru-proyecciones-de-poblacion-total-segun-departamento-provincia-y-distrito-2018-2022).

### Catchment Area Size for Each EFGH Country Site

The minimum number of confirmed *Shigella* cases at EFGH recruiting facilities and the size of the catchment area required to estimate *Shigella* incidence and 95% confidence intervals with specified precision in each EFGH country site are shown in Table 1. Assuming 5% of the population falls into the targeted age category, a catchment area population of ≥100 000 will be required for a 24 month recruiting period.

The minimum number of households needed for the population estimation survey and HUSs is based on the expected number required to estimate, with acceptable precision, the proportion of children aged 6–35 months with diarrhea brought for care to an EFGH health facility, based on 14 day recall. To achieve 500 children with diarrhea and a conservative expected 14 day diarrhea prevalence of 10%, HUSs will need to be conducted on ≥5000 children aged 6–35 months [7–9]. Assuming 10% of the catchment area falls within this age range and an average household size of 5 people, we expect 1 child per 2 households interviewed. Thus, ≥10 000 households will be visited over the 24 month period to achieve the required sample size.

### Cluster Size and Randomization

Catchment areas for participating health facilities were identified and mapped by EFGH country site teams. Each catchment area was subdivided into smaller clusters proportional to population density that could be randomly selected, enumerated by field teams, and, ultimately, provide an estimate of the entire catchment area population based on the proportion of enumerated clusters and their population.

The size and location of each cluster was determined using ≥1 grid square identical to the raster grid used by WorldPop. In WorldPop (www.worldpop.org), each grid square includes an estimate of its population size which was then used to optimize the size of the clusters in each catchment area. The optimal cluster size was determined to be 500 people per average per cluster, as it would allow a single field team to enumerate 1 cluster in approximately 1 week. With this cadence, each country site would enumerate the entire EFGH target population of approximately 100 000 people within a 24 month surveillance period.

**Figure 1.**
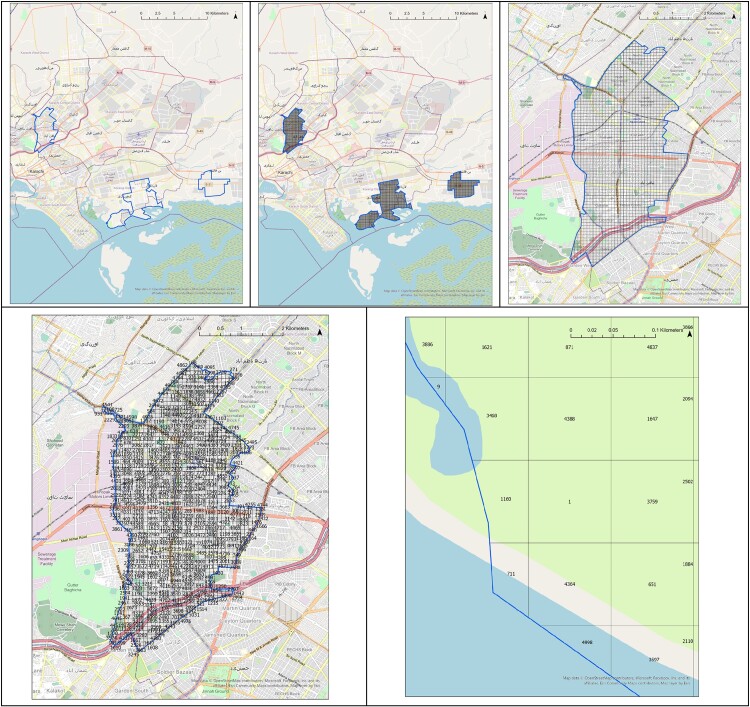
The process of creating and ordering clusters and assigning the cluster identification (ID) number within each Enterics for Global Health (EFGH) study area. *Top left,* EFGH-Pakistan study area borders (*bold line*). *Top center,* Square grid matching the dimensions of the Pakistan WorldPop raster (*grid of fine lines*), bounded by the EFGH-Pakistan study area (*bold line*). *Top right,* Detailed map of the northwest section of the Pakistan study area, with square grid (*grid of fine lines*) bounded by the EFGH-Pakistan study area (*bold line*). *Bottom left,* Each cluster is randomly numbered and labeled with a cluster ID number. Field teams visit each cluster in sequential order starting with cluster 1. Borders of the EFGH-Pakistan study area are bounded in blue. *Bottom right,* Detailed view of cluster 1 and surrounding clusters. The EFGH-Pakistan study site is bounded in blue. Cluster 711 is truncated by the study area. The Pakistan study area extends into the water in places owing to the delineation of official Union Council boundaries and the possibility of people living directly on the shoreline.

First, in each catchment area a vectorized square grid was overlaid onto the WorldPop constrained individual country population count raster in ESRI ArcGIS Pro Version 3.1.1 [10]. The WorldPop raster was then spatially joined with the overlaid grid so that each grid square had an associated population estimate (Figure 1). For a grid square that was truncated or crossed the boundary of a catchment area, an adjusted population estimate was calculated to account for the proportion of both the grid square and the population estimate within the boundary of the catchment area.

**Figure 2.**
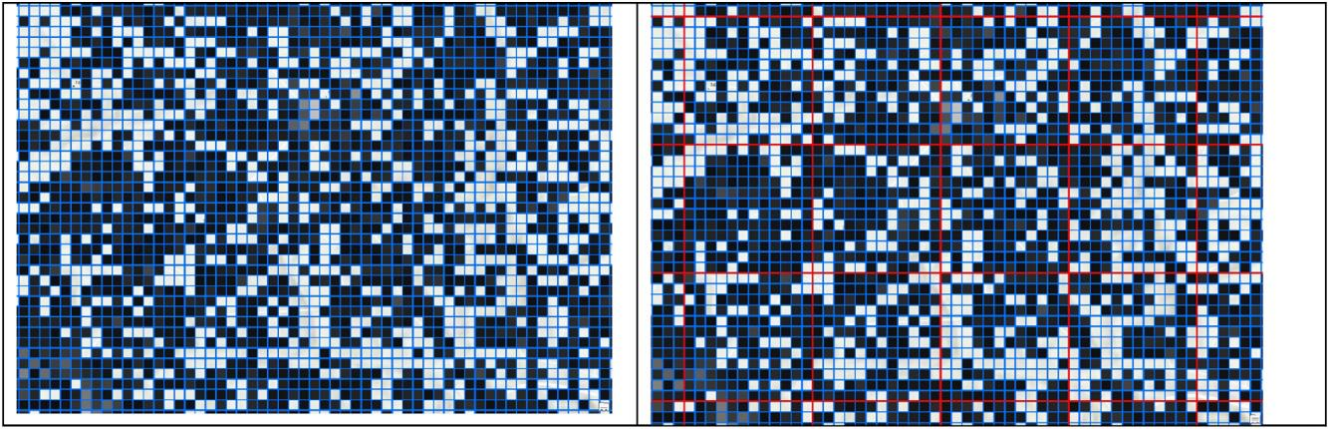
Using WorldPop to inform the size and estimated population within each cluster. *Left,* Square grid overlaid on the WorldPop raster in the Enterics for Global Health––Kenya study site. In this example, the average grid square has an estimated population of approximately 3.5 persons. *Right,* A total of 144 grid squares (*fine blue lines*) were combined to reach an average estimated population of 500 people per cluster (*bold red lines*). It was determined that clusters should be composed of 12 grid squares by 12 grid squares after iteratively calculating the optimum size using the equation, (500/*p*) × No. of input cells, where *p* is the mean population of input cells (which can be WorldPop grid squares or clusters from a prior iteration of the calculation). To ensure that clusters have a square aspect ratio, we took the square root of the number of required input cells and rounded it to the nearest whole number.

Each grid square was then given an identification (ID) number and a table containing the grid square ID number, grid square area (approximately 100 × 100 m^2^ for sites at the equator and 80 × 80 m^2^ for other sites) unless truncated by the catchment area boundary, and a WorldPop population estimate was generated. This table was used to calculate the average population of all grid squares with an estimated nonzero total population. Next, grid squares were combined to create clusters with 500 people per average adjusted population, to the nearest integer. In sites where the average adjusted population was equal to 500, grid squares were not combined. Instead, each square was considered its own cluster. Clusters with their associated population estimates were created for each catchment area (Figure 2) and assigned a cluster ID number.

**Figure 3.**
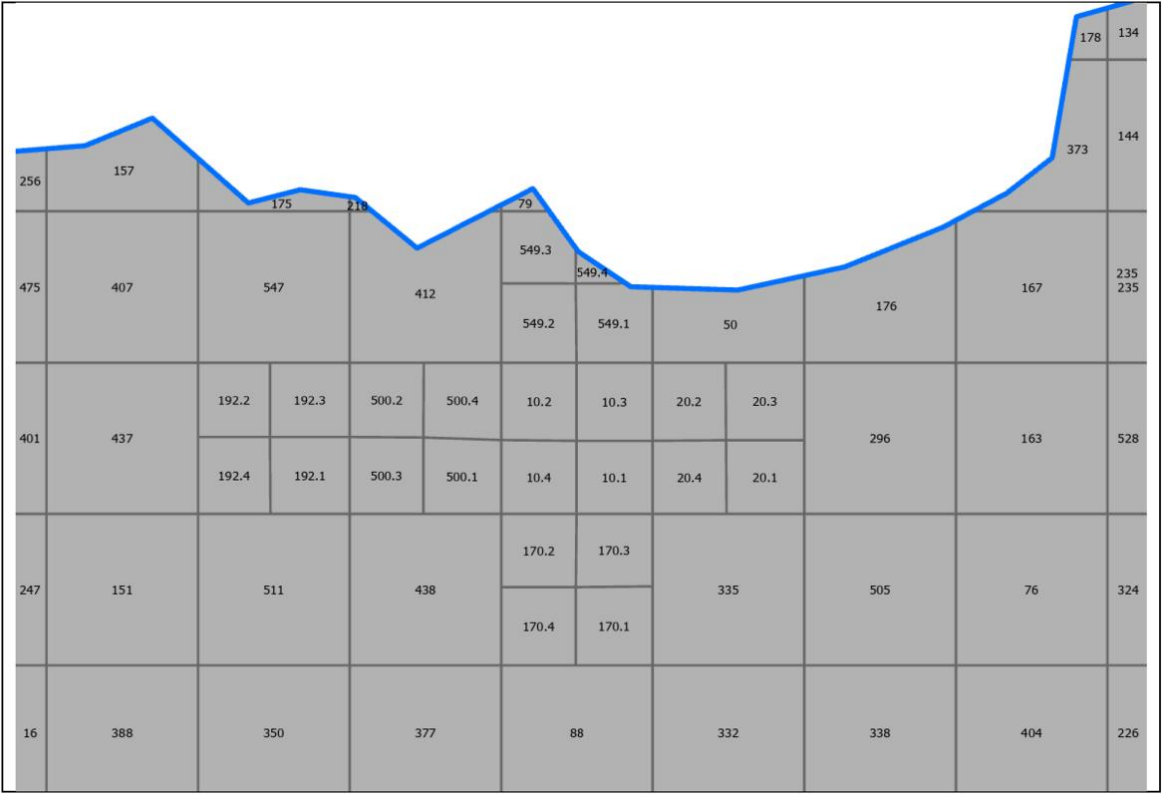
Subclusters (clusters 10, 170, 192, 500, and 549) in the Enterics for Global Health–Kenya study site, divided into quadrants based on estimated population size. Subclusters are shaded. Clusters along the boundary of the catchment area (*thicker boundary line*) are truncated; clusters 157 and 175 are examples of truncated clusters.

As some sites (e.g., The Gambia and Kenya) have substantially heterogeneous population densities across catchment areas, targeting an average 500 people per cluster permitted some clusters to have a substantially higher population. To make the size of these clusters more manageable, all primary clusters with a total estimated population of >2000 people were subdivided into 4 square subclusters of equal area. These subclusters were treated as a single cluster and assigned a single cluster ID number during randomization (Figure 3).

Finally, a table containing ID numbers of each primary cluster was randomly ordered and assigned a cluster ID number. The table was randomized in R software [11] and assigned a cluster ID number that was then reassociated with its cluster feature class by its original feature class ID number in ArcGIS Pro [10]. The feature class was then exported as a shapefile to be used imported onto tablets that would be used by field workers during enumeration activities.

### Population Enumeration

A population enumeration survey will be used to estimate the number of children aged 6–35 months, the age range of EFGH-included case patients [12], in each catchment area of participating EFGH health facilities. Field teams will systematically approach each household in the randomly assigned cluster. Before administering the population enumeration survey, field staff will identify the head of household or primary caregiver who is of legal age to consent. Field staff will then read a verbal script explaining the purpose of the EFGH study and the population enumeration survey. Consent forms have been approved by the ethical review board at each respective EFGH country site.

After obtaining verbal consent, field staff will administer the case report form using SurveyCTO [13] to determine the number and age of each child <5 years old in the household, the relationship of each child <5 years old to the head of household or primary caregiver, the date of birth of each child <5 years old, and whether the child has had diarrhea (≥3 abnormally loose or watery stools within a 24-hour period) during the previous 14 days. A household is defined by each site’s unique context, adapted from this basic definition: a group of people who share the same residence as permanent residents (not guests) and who either acknowledge the authority of the same head of the household and who eat from the same cooking pot. Household members will be defined by each site’s unique context, adapting from this definition: individuals who shared the same residence for ≥7 of the last 14 days as permanent residents (not guests). If no member of the household is present at the first attempt of household enumeration, the field team will make 2 additional attempts (3 enumeration attempts in total) to revisit the household for enumeration within 7 days of the initial visit.

### HUS Protocol

If during the population enumeration survey the primary caregiver reports that ≥1 child aged 6–35 months living in the household has had diarrhea in the previous 14 days, the HUS will be administered. Informed consent will be requested before administering the HUS. Should an enumerated household have an eligible child, but no appropriate household member is present to provide consent or participate in the HUS, a revisit will be scheduled.

Field staff will administer a standardized case report form in a password-protected mobile application, SurveyCTO, to ascertain information concerning recent healthcare-seeking behavior for diarrhea and dysentery in the last 14 days among children <5 years old. Field staff will identify the primary caregiver or household member best able to recall symptoms related to the most recent diarrheal episode for each child and will use the 14-day recall process, accompanied by visual aids, to help the caregiver remember details of any diarrheal episode. For each child aged 6–35 months who has experienced an episode of ≥3 abnormally loose or watery stools in a 24-hour period in the previous 14 days, the caregiver will be asked additional questions to further classify the diarrheal episode. Information will be sought regarding the presence of visible blood in the stool, illness severity (eg, signs of dehydration, duration, or vomiting), whether and where care was sought, and which interventions, if any, were administered to the child (ie, zinc, oral rehydration solution, antibiotics).

A site-specific wealth index will be administered to capture data regarding water, sanitation, hygiene practices, and indicators of socioeconomic status to further elucidate differences between households that did and those that did not seek care for diarrhea. The case report forms, visual aids, and wealth indices are available on Clinicaltrials.gov (NCT06047821).

### Field Surveys

Field teams will visit clusters sequentially when performing population enumeration and HUS activities. Field teams are given the complete list of randomized clusters in each catchment area to enable the EFGH country sites to survey households at a consistent pace during the 2-year surveillance period and have the flexibility to enumerate more than the target population of 100 000 people.

To identify clusters for enumeration, field teams will use Locus GIS [14] on handheld tablets to display shapefiles with clusters in the catchment area. Locus GIS will be used to identify the location of the randomized cluster, to confirm the cluster ID number and boundary, and to navigate clusters in the catchment area. Owing to limited internet access in rural and remote catchment areas, Locus GIS's offline mapping and location-viewing feature will be used.

Once at the household, field staff will collect population enumeration and HUS data using SurveyCTO [13]. SurveyCTO is a general data protection regulation (GDPR)-compliant, secure, and scalable mobile data collection platform with offline settings, featuring password protection with multiple layers of encryption and redundancy, ensuring end-to-end data encryption and access restricted solely through an encryption key from handheld devices to online servers. The database will be installed on tablets and laptops at each site and data will be collected in real-time. At the end of each interview or day (depending on availability of internet connection), country site staff will upload data to a central online secured server hosted at the University of Washington, Seattle. If an assigned cluster is considered to have no households, as determined through local knowledge, and confirmed using satellite imagery or confirmed by a physical visit to the cluster, the field team will record the cluster as completed with zero population.

### Field Team Training

Two representatives from each EFGH site attended a 4-day training-of-trainers workshop in Nairobi, Kenya, on population enumeration and HUS methods. Participants underwent training on standard operating procedures and administration of case report forms, and they engaged in mock sessions for cluster and household identification using Locus GIS [14] and SurveyCTO. On return to their respective EFGH sites, each attendee led a site-level training on the methods for sampling households within the catchment area, use of Locus GIS, and administration of case report forms using SurveyCTO.

Before study initiation, each site conducted a pilot that included obtaining consent and administering paper case report forms to 10 volunteer households outside the participating health facility’s catchment area. The completed case report forms were reviewed centrally to ensure standardization across sites.

### Quality Assurance and Quality Control Activities

At the end of each day, field staff will submit their tablets to site supervisors, who will verify the total number of households visited and total number of households enumerated per the log sheet. Feedback will be provided to the field staff if any issues are identified. Field staff and site supervisors will keep a log of households that need to be revisited in the next 7 days.

Data will be synchronized to the central SurveyCTO server daily. Data managers at each site will have access to the uploaded data to perform site-specific queries. The EFGH central data management team will generate monthly data queries and send a list of queries to site data managers. Alongside queries, country site data managers will perform data query checking based on site-level data quality assurance plans developed by each site.

## CHALLENGES AND PROPOSED SOLUTIONS

The use of hybrid surveillance methods to estimate the incidence of *Shigella* diarrhea is a strength of the EFGH study. However, there are several challenges in using population enumeration and HUS methods to estimate diarrhea incidence.

Obtaining an accurate incidence estimate requires that the case patients enrolled at EFGH health facilities reside in a defined catchment area and that the underlying population from this catchment area that will be used as the denominator to calculate incidence is representative of the cases. It is essential that EFGH health facilities have well-known (eg, political or administrative) geographic boundaries to accurately delineate the catchment areas and allow site staff to easily identify case patients residing within these boundaries. Strategies that have been used to inform catchment area boundaries for EFGH health facilities include retrospective geospatial plotting of the addresses of inpatient or outpatient case patients presenting to the facility [14], geolocation (GPS coordinates) of the addresses of prospectively identified case patients [15], and use of a catchment area within a demographic surveillance system [7, 8]. In the current study, we are using paper maps at some enrollment facilities to verify residence within the catchment area, which may result in some misclassification if screened individuals cannot accurately identify the location of their home.

An implicit assumption of facility-based hybrid surveillance for incidence estimation is that children with diarrhea brought for care at a given health facility are representative of children with diarrhea who are brought for care elsewhere or not at all [7, 8]. Household wealth and disease severity have been shown to influence care-seeking behavior in enteric fever and diarrheal studies [15, 16]. Similarly, we may expect that diarrhea cases in the community are less severe than those in patients who seek care at a health facility. Because the pathogenic etiology of diarrhea is associated with disease severity, a difference in severity between facility and community cases may result in a distorted distribution of diarrhea etiology. Similarly, age is associated with diarrhea etiology [7, 8, 16]. If children with diarrhea brought for care are systematically younger than children with diarrhea in the community, the etiologic distribution may be biased toward causes that are more common in younger children. EFGH will use data from the HUS to adjust for differences in care-seeking behaviors, but if these factors are not appropriately accounted for, the incidence estimates could be biased.

Population fluctuations (e.g., seasonal migration and displacement following severe weather conditions) and disease outbreaks sharing similar clinical manifestations as *Shigella* infection during the study period can affect population enumeration and healthcare utilization estimates, potentially introducing bias. To reduce this bias, EFGH will use a sampling strategy that enumerates households within randomly assigned, geographically diverse clusters in each EFGH catchment area continuously throughout the 2-year study period, alongside diarrhea case surveillance at EFGH health facilities. This ensures representative sampling of the population at risk throughout the period of enrollment.

## CONCLUSIONS

In the EFGH study, the methods used for population enumeration and HUSs leverage the advantages of each method above while minimizing the disadvantages. The methods will use an efficient sampling approach that will account for poor parity between survey area coverage and facility coverage, population fluctuations, seasonal variability, and adjustments to care-seeking behavior. The rigorous surveillance methods in EFGH will be used to generate reliable estimates of population-based diarrhea in children aged 6–35 months and will help inform future *Shigella* vaccine trials.

## Notes

***Author contributions.*** R.D., A.O.A., P.F.G.B., F.K., D.M., U.O., G.S., and M.T.Y (with review, scientific input, and editing from N.A.C., M.J.H., R.O., F.N.Q., M.D.T., P.P.Y., K.Z., and C.J.M) wrote the first draft of the manuscript and actively participated in monthly working group meetings. N.A., H.E.A., M.A.I.B., J.M.C., B.C., M.D., N.D., S.E., I.F., E.F., S.R.G., S.H., M.T.I., M.K., B.K., M.N.K., K.L.L., C.L., M.M., M.N., L.N., B.O., C.O., M.P.O., P.B.P., T.P.V., J.A.P., F.Q., S.Q., E.T.R.M., S.S., and M.O.T. reviewed and edited the manuscript. All authors approved the content of the final manuscript.

***Disclaimer.*** The views expressed are those of the authors and not necessarily those of the National Institute for Health and Care Research, the Department of Health and Social Care, the UK government, or the UK Health Security Agency.

***Financial support.*** This work was supported by the Bill & Melinda Gates Foundation (grants INV-016650, INV-031791, INV-036891, and INV-36892) and the National Institutes of Health (grants D43TW010913 to M. N. K. and M. P. O. and 1K01AI168493-01A1 to J. M. C.). The Gambia team's work is also supported by the UK Research and Innovation Medical Research Council (program MC_UU_00031/1—Disease Control and Elimination). N. A. C. is a National Institute for Health and Care Research (NIHR) senior investigator (NIHR203756); he is affiliated with the NIHR Global Health Research Group on Gastrointestinal Infections at the University of Liverpool and with the NIHR Health Protection Research Unit in Gastrointestinal Infections at the University of Liverpool, a partnership with the UK Health Security Agency in collaboration with the University of Warwick.

***Supplement sponsorship.*** This article appears as part of the supplement “Enterics for Global Health (EFGH) *Shigella* Surveillance Study-Rationale and Methods,” sponsored by the Bill & Melinda Gates Foundation.
